# Clinical and programming pattern of patients with impending deep brain stimulation power failure: a retrospective chart review

**DOI:** 10.1186/2054-7072-1-6

**Published:** 2014-11-20

**Authors:** Raja Mehanna, Kathy M Wilson, Scott E Cooper, Andre G Machado, Hubert H Fernandez

**Affiliations:** University of Texas Health Science Center, 6410 Fannin Street, Suite 1014, Houston, TX 77030 USA; Cleveland Clinic Foundation, Cleveland, Ohio USA

**Keywords:** Deep brain stimulation, Estimate, Depletion

## Abstract

**Background:**

It is important to prevent complications of implanted pulse generators (IPG) depletion by replacing the IPG in time.

**Methods:**

We reviewed the charts of all patients with deep brain stimulation treated movement disorders who were seen at our institution over a period of 6 months. Among these, we retained for analyses those who had undergone IPG replacement within the previous 3 years.

**Results:**

A total of 55 IPG replacements (from 38 patients) were reviewed. Electrodes were implanted in the subthalamic nucleus in all Parkinson’s disease patients, in the ventral intermedius nucleus of the thalamus in all essential tremor patients and in the globus pallidus interna in all dystonia patients. Replacements were preceded by a voltage increase due to worsened symptoms in 27.3% (15/55); 25.5% (14/55) had full IPG depletion or had too low IPG reserve to allow for any voltage adjustment; and 21.7% (12/55) did not get a needed voltage increase either for safety reasons (eg: concern for increase in falls with higher voltages) or because the surgery date for IPG replacement was close. Only 25.5% (14/55) remained clinically well-controlled prior to IPG replacement, all of whom had IPG longevity estimates available. Clinical deterioration was noted prior to IPG replacement in 100% of patients without available longevity estimates versus 61% of patients with available longevity estimates (p < 0.001).

**Conclusion:**

Despite best efforts, clinical deterioration prior to IPG replacement can be seen frequently. Routine estimation of IPG life, along with symptom assessment at every follow-up visit may prevent it.

**Electronic supplementary material:**

The online version of this article (doi:10.1186/2054-7072-1-6) contains supplementary material, which is available to authorized users.

## Background

Deep brain stimulation (DBS) has been approved by the United States Food and Drug Administration (FDA) for the treatment of Parkinson’s disease (PD), essential tremor (ET) and dystonia, and is undergoing trials for approval in psychiatric disorders such as depression and obsessive compulsive disorders.

DBS acts through delivering an electrical current in a specific target area of the brain, the target being different according to the disease being treated. This current can be modulated through modification of voltage, frequency and duration of each electrical pulse delivered. The delivered energy creates an electrical field of variable size and shape according to the parameters used for stimulation [1]. The current is generated by an implantable pulse generator (IPG), a small pacemaker-like unit that is implanted under the skin, usually in the chest or less frequently in the abdomen. The current is then delivered through an extension wire and implanted electrode to the target located deep in the brain. Once the IPG is depleted, it has to be replaced so current can continue to be generated and delivered to the brain.

Because IPG depletion can result in worsening of neurological symptoms [2, 3], and sometimes lead to medical emergencies [4, 5], it is important to prevent it by replacing the IPG in time. This can be done by routinely estimating IPG life and assessing symptoms at every follow-up visit. For the Soletra model, estimate can be done by telephoning Medtronic technical support. Reported DBS settings are entered into a computer software which estimates the longevity of the IPG from the date of implant. A web based application developed by the University of Florida is also available [6]. However, the estimation of IPG life is always an approximation because the actual IPG life depends upon multiple specific DBS treatment parameters, duration of stimulation, lead impedance, as well as many other factors that cannot easily be estimated [7]. While increasing DBS settings correlates inversely with IPG longevity [8], guidelines provided by medical device companies to help approximate the longevity of an IPG cannot take into consideration all possible therapeutic combinations of DBS and are thus far from accurate, especially because DBS treatment parameters change over time with the evolution of the underlying disease. There are many more sources of error that may flaw longevity estimation, including but not limited to device-to-device variation, decreased supplied voltage with battery usage, battery chemistry, impedance fluctuation and battery self-discharge through quiescent current [7].

There is little information in the literature regarding practices when DBS batteries are about to be depleted of power. We reviewed all IPG replacements done at our institution over a period of 42 months to survey our practice patterns, including DBS programming modifications, when DBS batteries are nearing end of life.

## Methods

We reviewed the charts of all patients with movement disorders who underwent DBS surgery and who were seen at the Center for Neurological Restoration at Cleveland Clinic between 06/01/2012 and 12/31/2012. We retained for analyses those who had undergone IPG replacement within the previous 3 years. All batteries replaced being of the Soletra model (Medtronic ® Minneapolis, MN, USA), we included all replacements that were done for an IPG voltage of 3.69 or less, or for a drop by 0.3 V or more in the previous 12 months, or for clinical worsening that improved after IPG replacement, or at the family’s insistence because the predicted lifespan was reached. We excluded the IPG replacements that were done for other reasons (e.g. replacing the non-depleted contralateral IPG to spare the patient another surgery within a year). The patient’s age, gender, diagnosis, duration of disease at time of IPG replacement, IPG model and voltage at the last visit before IPG replacement were noted. The presence of symptoms attributed at the time by the treating clinician to IPG depletion and programming changes to address these symptoms were also recorded, as well as whether the IPG longevity estimate was known at the last visit before replacement, obtained through the Medtronic helpline. This was a minimal risk study utilizing existing data through chart review and not requiring any direct patient evaluation for the purpose of the study. Data was de-identified, informed consent was waived and the study was submitted to the Cleveland Clinic Foundation Institutional Review Board who exempted it from review.

## Results

**Figure 1:**
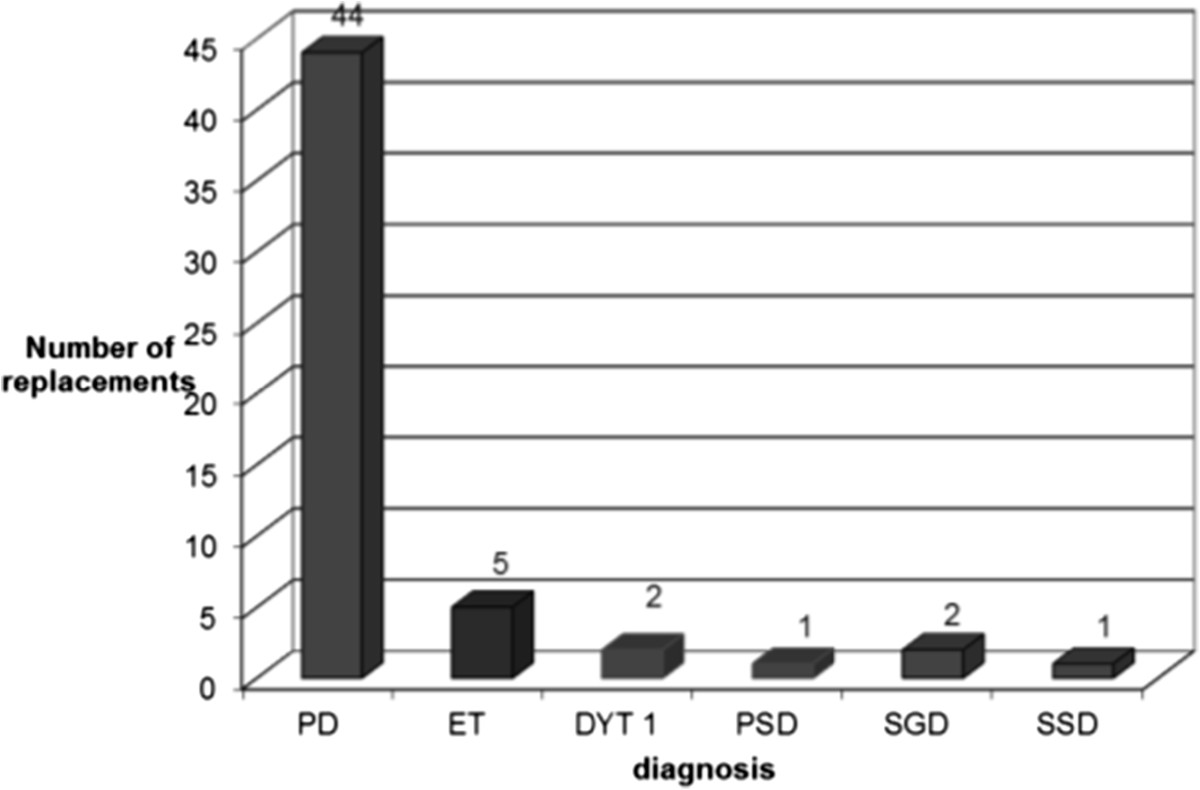
**Diagnosis distribution.** *Legend:* PD: Parkinson’s disease, ET: essential tremor, DYT 1: generalized dystonia with DYT 1 mutation, PSD: primary segmental dystonia, SGD: secondary generalized dystonia, SSD: secondary segmental dystonia.

**Figure 2:**
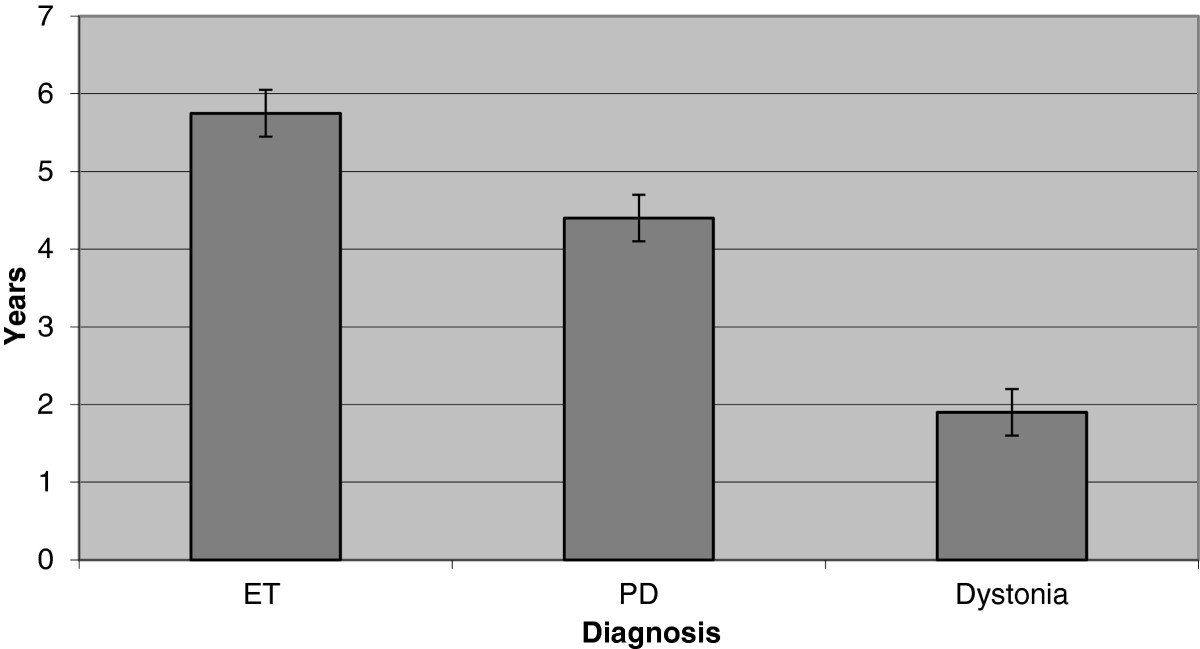
**IPG longevity per diagnosis in years.** *Legend*: IPG: implantable pulse generator, PD: Parkinson’s disease, ET: essential tremor.

A total of 55 IPG replacements, involving 38 patients, with 25 (66%) males and a mean age of 67.8 years (range 23 to 90 years), were ultimately included in this survey (Table 1). The diagnoses included PD (44 replacements), ET (5 replacements), primary generalized dystonia with DYT 1 mutation (2 replacements), primary segmental dystonia (1 replacement), secondary generalized dystonia (2 replacements) and secondary segmental dystonia (1 replacement) (Figure 1). All PD patients had their electrodes implanted in the subthalamic nucleus; ET patients had their electrodes implanted in the ventral intermedius nucleus of the thalamus; and all patients with dystonia had theirs implanted in the globus pallidus interna. On average, the batteries were 4.3 years old (range 1.2 – 9 years) and had a mean voltage of 3.39 (range 0 – 3.74 V) when they were replaced (Table 1). When assessing IPG longevity by diagnosis, the mean lifespan was 5.75 years in ET (range 4–6.75 years, SD 1.09, SEM 0.5), 4.4 years in PD (range 1.5-9 years, SD 2.02, SEM 0.3) and 1.9 years in dystonia (range 1.2 to 3 years, SD 0.73, SEM 0.3)(F = 6.451, p = 0.003) (Figure 2).

**Table 1:** **Patients characteristics**

	Average	Range
Age	67.8 years	23–90 years
M/F ratio	2/1	
Age of battery when replaced	4.3 years	1.2–9 years
Battery voltage when replaced	3.39 V	0-3.74 V

**Figure 3:**
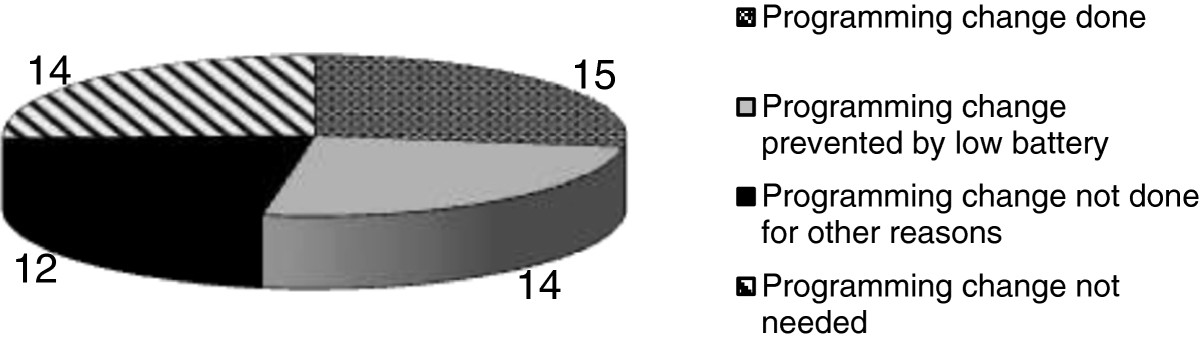
**Programming change prior to surgery.**

In 15 of 55 (27.3%) replacements, patients required a voltage increase due to worsened symptoms (mean voltage increase = 0.26 V; SD = 0.1; range: 0.1-0.5 V) (Figure 3). This increase at least partially improved the symptoms until the IPG was replaced. Moreover, for these patients, the side effects noted *after* IPG replacement often required voltage re-adjustment back to their pre-power depletion levels. In 14 (25.5%) additional replacements, a needed voltage adjustment could not be performed because of either full IPG depletion or too low IPG reserve, with the concern that such increase would precipitate depletion before replacement. Additionally, 12 (21.7%) replacements did not get a needed voltage increase either for safety reasons such as concern for increase in falls with higher voltages or because patient declined it as surgery date was close (Figure 3). Only 14 of 55 (25.5%) replacements were clinically well-controlled prior to IPG power depletion (Figure 3). Their IPGs were replaced well ahead of full depletion and no programming adjustments were necessary.

In 33 of 55 (60%) of the replacements, the IPG longevity estimate was available. Twenty of these (61%) had motor deterioration before IPG replacement, 5 of which benefited from a voltage increase prior to IPG replacement. 6 replacements were done at the patient’s and family insistence based on previous estimates, despite good IPG reserve and the lack of symptoms of IPG depletion. In contrast, all the 22 replacements that did not have estimates available had symptoms of depletion before IPG replacement. The difference in the proportion of patients who experienced worsening of symptoms prior to IPG replacement between the group with estimates available (61%) versus the group without available estimates (100%) was statistically significant using a Chi square test (Chi-square = 11.35; p < 0.001).

## Discussion

In our retrospective review of 55 IPG replacements in 38 patients, the most frequent diagnosis was PD (76.3%), followed by dystonia (13.2%) then ET (10.5%). We have found that the average longevity of the Soletra IPG was consistent with previous reports for patients with dystonia at 1.9 years [9–12] and PD at 4.4 years [6, 8, 13, 14]. However, the IPG lasted longer that previous reports in our patients with ET (5.75 vs 2 to 4 years) [6, 8, 12, 13], likely because most of our ET patients turn their IPG off at night precisely to prolong its life. Overall, this was consistent with previously published data that the underlying diagnosis can also affect IPG longevity with the characteristics of the stimulation target as well as the disease pathophysiology likely contributing to battery longevity [6, 12, 13], with dystonia typically depleting the IPG faster than PD or ET.

Clinical deterioration prior to IPG replacement occurred frequently. Fifteen of 55 (27.3%) replacements benefited clinically from a voltage increase (mean voltage increase = 0.26 V; SD = 0.1; range: 0.1-0.5 V). Interestingly, side effects noted after IPG replacement often required a voltage re-adjustment to their pre-power depletion levels. In 14 of 55 (25.5%) replacements, patients were clinically worse but could not get a needed voltage increase because of either full IPG depletion or too low IPG reserves. Finally, 14 of 55 (25.5%) IPGs were replaced well ahead of full depletion and no programming adjustments were necessary before or after IPG replacement. All of the latter had IPG longevity estimates available. We believe that knowing these longevity estimates prevented clinical deterioration prior to IPG replacements in several patients in our cohort—clinical deterioration was noted in 100% of IPG replacement without longevity estimates, whereas it was seen in 61% of IPG replacements with available longevity estimates (p < 0.001).

Available data regarding the clinical usefulness of longevity estimate are conflicting. In a prospective study on 72 IPG replacements, Stewart and Eljamel [15] demonstrated large differences between the actual longevity and the longevity predicted using a computer-based estimate integrating current DBS parameters. There was no correlation between the 2, and the actual longevity was shorter by a year in some instances. However, in a retrospective review of 320 charts, Fakhar et al. demonstrated that an University of Florida web based estimator/smart phone application (r = .67, p < .001) as well as the Medtronic helpline estimate (r = 0.74, p < 0.001) were correlated with the actual IPG life [6].

Our study replicate the results from Fakhar et al., albeit on a smaller sample and without the use of the University of Florida estimator. These studies actually complement each other with an emphasis on the physical and electrical aspect in Fakhar et al.’s, and on the clinical aspect in ours. The IPG longevity in ET patients was, however, longer in our study (5.75 vs 3.54 years). In our study, knowledge of the estimate was associated with a lower rate of symptomatic IPG depletions. However, because only 4 of the 55 IPGs were totally depleted at the time of surgery, we could not compare the estimated lifetime to the actual lifetime of the IPGs.

**Figure 4:**
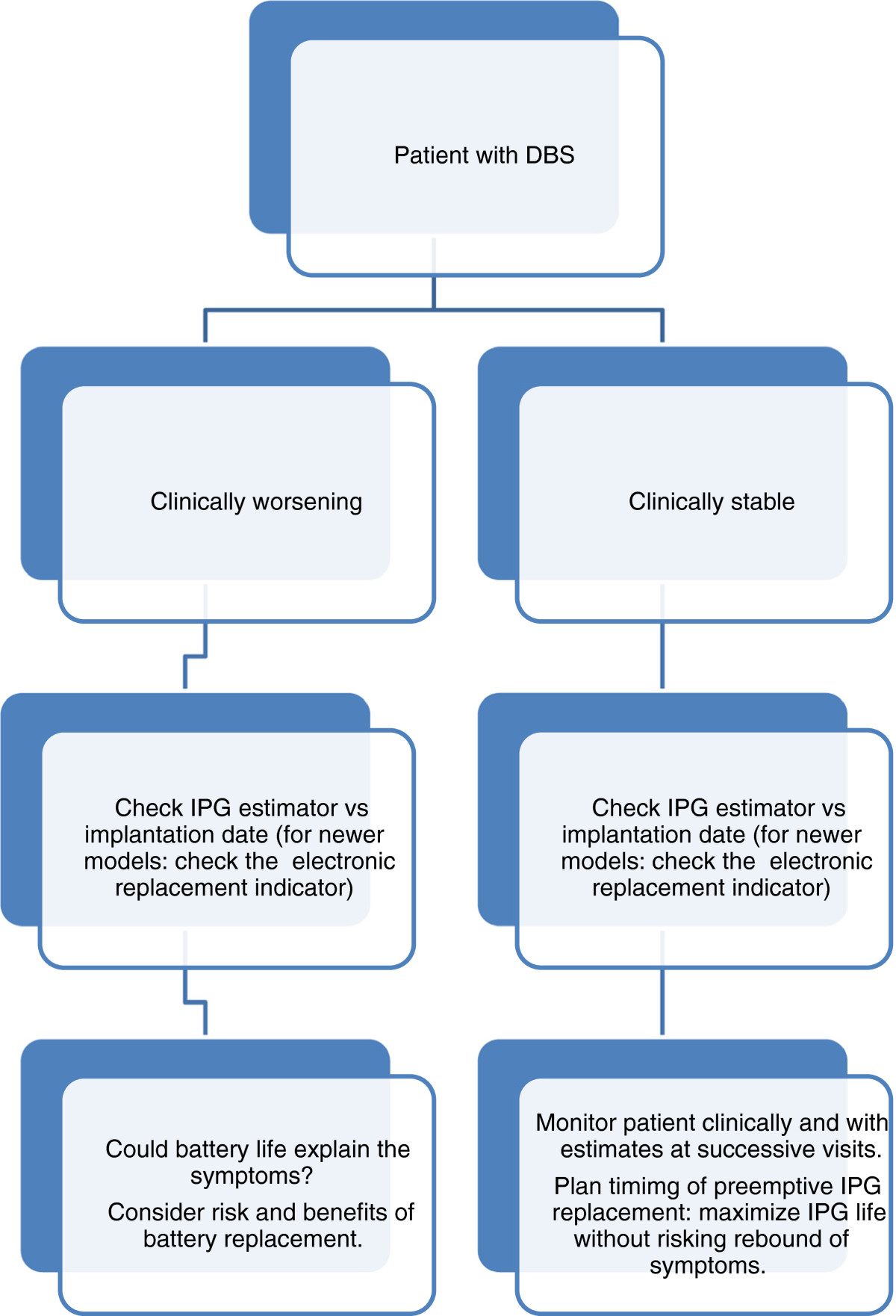
**Suggested algorythm for management of IPG life.** *Legend*: IPG: implantable pulse generator.

While this study has some limitation inherent to its small size and retrospective design such as reliance on recorded data and chart quality, as well as lack of randomization, we believe it is a useful survey of practice patterns at our institution and suggests that IPG longevity estimate is useful in clinical practice. Considering that 6 of 55 (11%) of replacements in our study were done at the patients or family insistence because the predicted lifetime was coming to an end, although there were no clinical or electrical signs of depletion, the question of cost effectiveness of relying solely on longevity estimate should be raised. However, this contrasts with previous reports where the actual life expectancy was typically shorter than estimated [15]. It thus seems that both the estimate as well as the electrical and/or clinical worsening should be taken into consideration when deciding of the best time to replace the IPG, supporting an algorithm previously suggested by Montuno et al. (Figure 4) [7].

Finally, all the patients in this study were found to have the Soletra model of IPG. This specific model is old and has been replaced by newer models such as Activa. The newer models have an elective replacement indicator message that is displayed on patient and clinician programing devices, and suggests that a replacement may be required within approximately three months. The newer models probably predict battery failure more accurately, and the results of our study might not apply to them. However, clinicians taking care of the non-negligible number of patients still carrying Soletra, and until these deplete and get replaced by a newer model, will find our results still useful and clinically relevant.

## Conclusions

The patients who had optimal symptom control were those seen well in advance of power depletion, with enough time to have batteries replaced before their symptoms worsened. However, clinical deterioration just prior to end of IPG life was not uncommon. A significant percentage of patients required voltage adjustments prior to IPG placement to improve their worsening clinical state, and often required another voltage readjustment after IPG replacement. Because IPG depletion can lead to worsening of neurological symptoms and even medical emergencies on one hand, and overzealous IPG replacement may not be cost-effective on the other, DBS programming clinicians should routinely estimate IPG life and assess symptoms at every follow-up visit in order to decide when IPGs should be replaced.

## Authors’ original submitted files for images

Below are the links to the authors’ original submitted files for images. Authors’ original file for figure 1 Authors’ original file for figure 2 Authors’ original file for figure 3 Authors’ original file for figure 4 Authors’ original file for figure 5

## Abbreviations

IPG: Implanted pulse generators
DBS: Deep brain stimulation
FDA: Food and Drug Administration
PD: Parkinson’s disease
ET: Essential tremor.
