# Supplementary figures and images for: Clinical and programming pattern of patients with impending deep brain stimulation power failure: a retrospective chart review
Source: J Clin Mov Disord. 2014 Nov 20;1:6. doi: 10.1186/2054-7072-1-6 (PMC4677734; doi:10.1186/2054-7072-1-6)

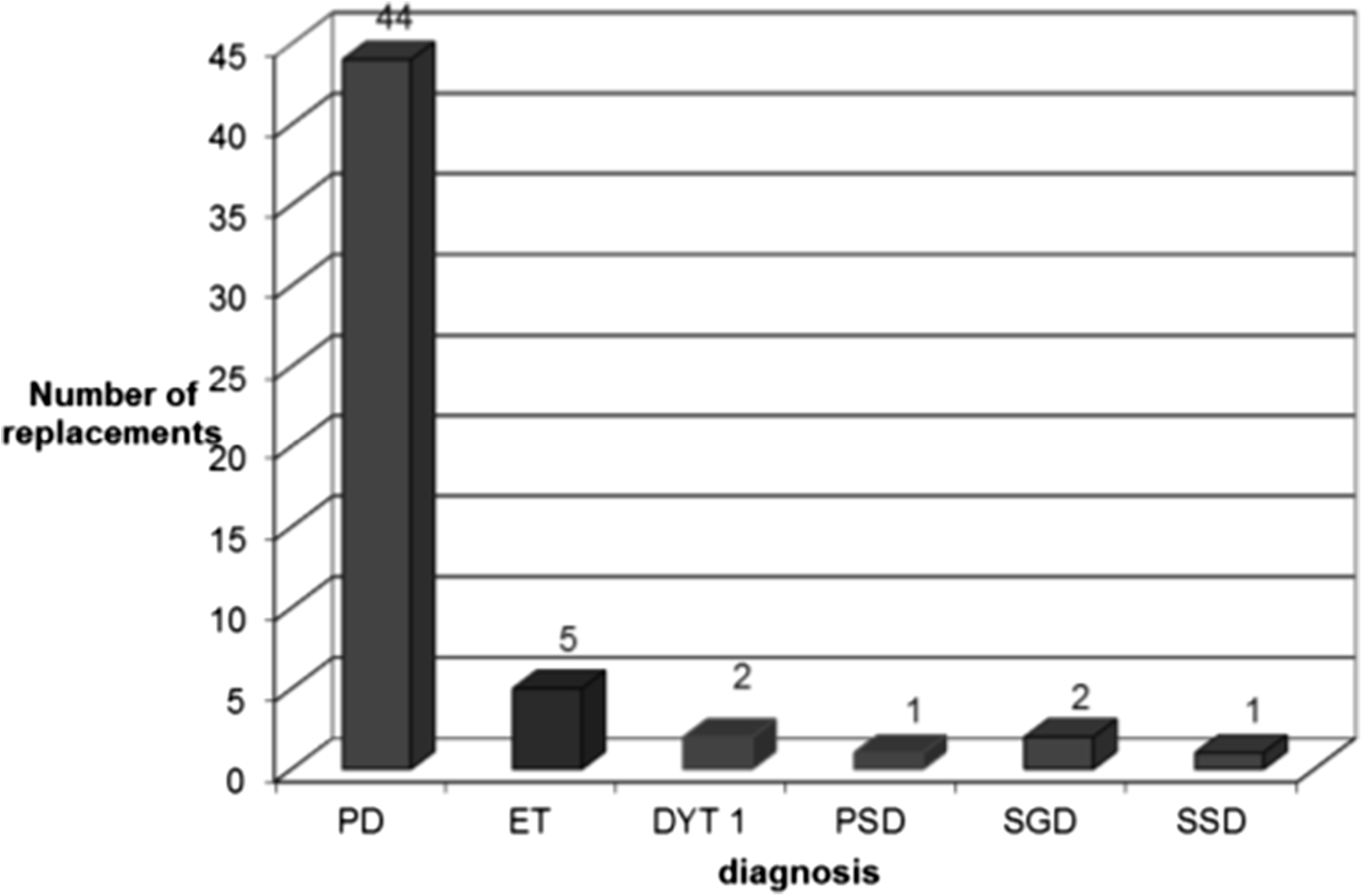

Supplement: Supplementary file 1 — Authors’ original file for figure 1 [file 40734_2014_8_MOESM1_ESM.tiff]

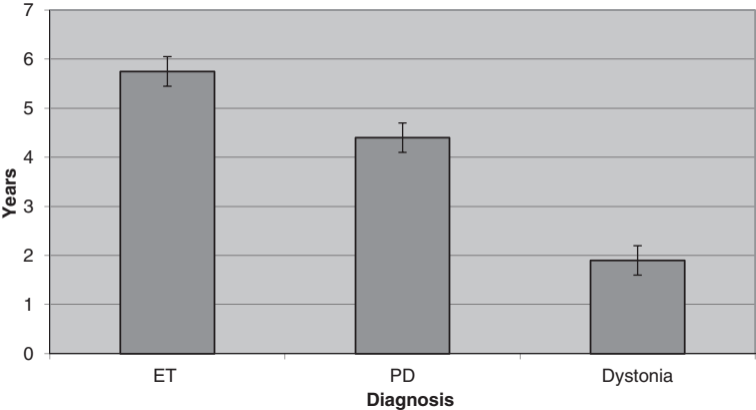

Supplement: Supplementary file 2 — Authors’ original file for figure 2 [file 40734_2014_8_MOESM2_ESM.pdf]
