# Supplementary material for: Clinical and programming pattern of patients with impending deep brain stimulation power failure: a retrospective chart review
Source: J Clin Mov Disord. 2014 Nov 20;1:6. doi: 10.1186/2054-7072-1-6 (PMC4677734; doi:10.1186/2054-7072-1-6)
Supplement: Supplementary file 3 — Authors’ original file for figure 3 [file 40734_2014_8_MOESM3_ESM.pdf]

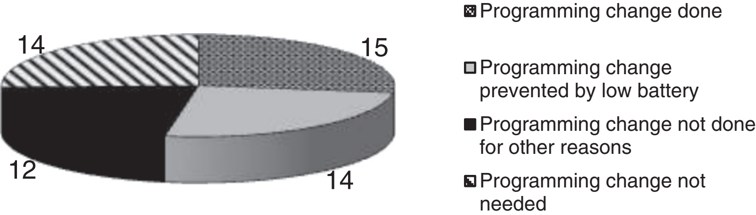
14
15
12
14
Programming change done
Programming change prevented by low battery
Programming change not done for other reasons
Programming change not needed
