# Supplementary material for: Clinical and programming pattern of patients with impending deep brain stimulation power failure: a retrospective chart review
Source: J Clin Mov Disord. 2014 Nov 20;1:6. doi: 10.1186/2054-7072-1-6 (PMC4677734; doi:10.1186/2054-7072-1-6)
Supplement: Supplementary file 4 — Authors’ original file for figure 4 [file 40734_2014_8_MOESM4_ESM.pdf]

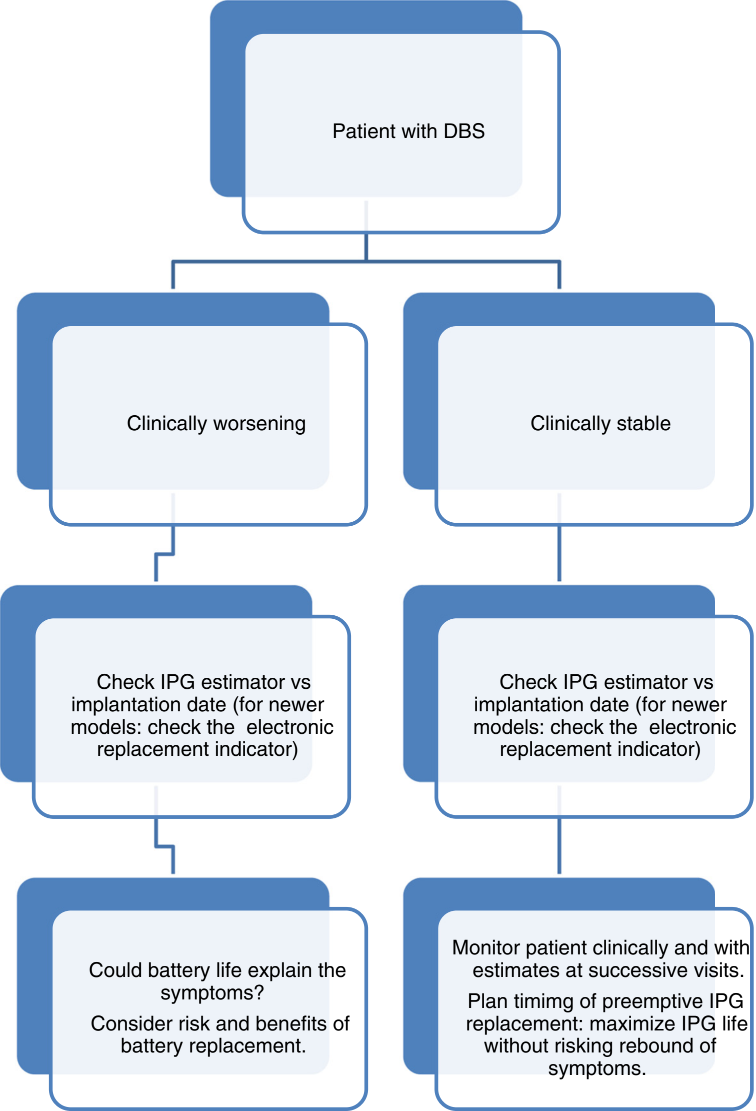
Patient with DBS
Clinically worsening
Clinically stable
Check IPG estimator vs implantation date (for newer models: check the electronic replacement indicator)
Check IPG estimator vs implantation date (for newer models: check the electronic replacement indicator)
Could battery life explain the symptoms?
Consider risk and benefits of battery replacement.
Monitor patient clinically and with estimates at successive visits.
Plan timimg of preemptive IPG replacement: maximize IPG life without risking rebound of symptoms.
