# Supplementary material for: Clinical and programming pattern of patients with impending deep brain stimulation power failure: a retrospective chart review
Source: J Clin Mov Disord. 2014 Nov 20;1:6. doi: 10.1186/2054-7072-1-6 (PMC4677734; doi:10.1186/2054-7072-1-6)
Supplement: Supplementary file 5 — Authors’ original file for figure 5 [file 40734_2014_8_MOESM5_ESM.docx]

**Table**: Patients characteristics.

|  | Average | Range |
| --- | --- | --- |
| Age | 67.8 years | 23-90 years |
| M/F ratio | 2/1 |  |
| Age of battery when replaced | 4.3 years | 1.2 – 9 years |
| Battery voltage when replaced | 3.39 V | 0-3.74 V |
